# ASO Author Reflections: Old Truths, New Data: Intraductal Oncocytic Papillary Neoplasm-Derived Pancreatic Cancer Requires Continued Surveillance

**DOI:** 10.1245/s10434-025-18007-z

**Published:** 2025-08-20

**Authors:** Camila Hidalgo Salinas, Joseph R. Habib, Ammar A. Javed, Greg D. Sacks

**Affiliations:** 1 Department of Surgery, NYU Grossman School of Medicine, New York, NY USA; 2 Nuffield Department of Primary Care Health Sciences, Centre for Evidence-Based Medicine, University of Oxford, Radcliffe Observatory Quarter, Oxford, UK; 3 Department for Continuing Education, University of Oxford, Oxford, UK; 4 Department of Surgery, Regional Academic Cancer Center Utrecht, UMC Utrecht Cancer Center & St. Antonius Hospital Nieuwegein, Utrecht, The Netherlands

## Past

Intraductal papillary mucinous neoplasms (IPMNs) are recognized precursors to pancreatic cancer, with invasive subtypes historically categorized as tubular, colloid, or oncocytic.^1^ Among these, intraductal oncocytic papillary neoplasm (IOPN)-derived pancreatic cancer is recognized as a distinct entity with unique histologic and genetic features and has long been thought to carry a more favorable prognosis. However, due to its low incidence, comprehensive data on its clinical behavior and long-term outcomes remain limited.

## Present

Recent conflicting reports have challenged the historical notion of favorable outcomes for IOPN-derived pancreatic cancers.^2,3^ This study, analyzing one of the largest cohorts of resected IOPN-derived cancers to date, reinforced the longstanding understanding of its indolent nature.^4^ The findings showed that IOPN-derived cancers are associated with longer overall survival and time to recurrence than tubular IPMN-derived cancers and outcomes comparable with those for colloid subtypes. These findings persisted even after adjustment for known adverse pathologic features. The IOPN-derived cancers were more frequently in an early stage, with lower rates of nodal involvement, poor differentiation, and perineural or lymphovascular invasion, further supporting a less aggressive biology. Nonetheless, recurrence occurred in approximately 25 % of patients, underscoring the importance of continued surveillance.

## Future

The findings in this study support the notion that IOPN-derived pancreatic cancers follow a more indolent disease course than other IPMN-derived subtypes. However, the persistent risk of both local and systemic recurrence highlights the need for long-term surveillance. The role of adjuvant therapy remains unclear and may need to be based on patient and tumor characteristics.^5^ Future multicenter collaborations with centralized pathology review and molecular profiling are essential to refine management strategies and deepen our understanding of this rare but important pancreatic cancer subtype.
